# Retraction: The gender and culture effect on the CO_2_ emission empirical analysis

**DOI:** 10.1371/journal.pone.0306373

**Published:** 2024-06-27

**Authors:** The PLOS ONE Editors

After this article [1] was published, concerns were raised about overlap between this publication and a previous article by another group [2] that was not cited in [1].

Members of the *PLOS ONE* editorial team confirmed a high degree of similarity between the articles. We noted overlap in the research question, data sources, methods employed, conclusions and overall structure of the two articles. Additionally, the results reported in [1] appear very similar to those reported in the tables of [2], although individual data values reported are not identical.

The corresponding author stated that there were several differences between the papers, including the tables and significance values reported. The author’s comments did not resolve PLOS’ concerns.

In reviewing this matter, PLOS identified additional concerns pertaining to the article’s authorship, references, and dataset.

In light of the above concerns, the *PLOS ONE* Editors retract this article.

YZ, KC, and CZ did not agree with retraction. YZ and CZ stand by the article’s findings.

Contents of the retracted article [1] were removed from the *PLOS ONE* website at the time of retraction due to the similarities to [2]. The article’s Copyright and Data Availability statements were also updated at the time of retraction, and the removed contents are no longer offered under the Creative Commons Attribution License.
